# *Trichophyton mentagrophytes* type VII: cohort study on patient characteristics, clinical features, disease course, and treatment

**DOI:** 10.1111/ddg.15837

**Published:** 2025-07-31

**Authors:** Konstanze Kämmerer, Julia Huynh, Cornelia Deutsch, Stefanie May, Sertac Uyar, Alexander Nast, Christoph Zeyen, Ricardo Niklas Werner

**Affiliations:** ^1^ Department of Dermatology Venereology and Allergology Division of Evidence‐Based Medicine in Dermatology (dEBM) Charité – Universitätsmedizin Berlin corporate member of Freie Universität Berlin and Humboldt‐Universität zu Berlin Berlin Germany; ^2^ Department of Dermatology Venereology and Allergology Charité – Universitätsmedizin Berlin corporate member of Freie Universität Berlin and Humboldt‐Universität zu Berlin Berlin Germany; ^3^ Department of Dermatology Venereology and Allergology Mycology Laboratory Charité – Universitätsmedizin Berlin corporate member of Freie Universität Berlin and Humboldt‐Universität zu Berlin Berlin Germany

**Keywords:** dermatophytosis, kerion celsi, Majocchi granuloma, sexually transmitted diseases, tinea, *Trichophyton mentagrophytes* genotype VII, terbinafine

## Abstract

**Background and Objectives:**

Sexually transmitted infections with *Trichophyton mentagrophytes* type VII (TMVII) have been reported in several countries. The aim of our study was to identify epidemiological and clinical features in order to improve prevention, diagnosis, and treatment.

**Patients and Methods:**

Patients with culture‐confirmed TMVII infection diagnosed between 2020 and 2024 at the Department of Dermatology, Charité – Universitätsmedizin Berlin, Germany, were included. Patient characteristics, clinical presentation, and treatment were assessed.

**Results:**

Fifty‐one patients were included (96.1% male; median age, 34 years), with 94.6% of male patients reporting male sex partners. Among 47 patients with known HIV status, 19.1% were living with HIV, while 71.1% of HIV‐negative patients reported using pre‐exposure prophylaxis (PrEP). Median time to diagnosis was 42.5 days (Q1–Q3: 29.25–73.5). Atypical morphologies, such as purulent or infiltrated subcutaneous lesions, occurred in 82.4%, and 96.1% had genital, perianal, or perioral manifestations. Median systemic treatment duration was 83.5 days (Q1–Q3: 57–110.25), with no significant differences by morphology (*p* = 0.69) or HIV/PrEP status (*p* = 0.88).

**Conclusions:**

TMVII infections often present with atypical morphology and should be considered in the differential diagnosis of genital, perianal, and perioral dermatoses, particularly among men with multiple male partners. Clinicians should anticipate prolonged treatment durations to achieve clearance and prevent recurrence.

## INTRODUCTION

Dermatophytes cause infections of the skin and its appendages that present with a wide spectrum of clinical manifestations. Tinea involving areas near mucous membranes – such as the genital, inguinal, perianal, gluteal, and perioral regions – has been associated with various dermatophyte species.^1^, ^2^, ^3^, ^4^, ^5^, ^6^, ^7^, ^8^, ^9^, ^10^ Whereas cases of autoinoculation from other body regions^4^, ^8^ or other exposures^6^, ^10^, ^11^ have been reported, infections at these anatomic regions suggest possible sexual transmission.^2^, ^3^, ^5^, ^7^

*Trichophyton mentagrophytes* (TM), a zoophilic dermatophyte often associated with inflammatory and purulent lesions, has been frequently reported in patients with suspected sexual transmission.^12^, ^13^, ^14^ In 2019, a genetic variant, TM genotype VII (TMVII), was identified as a distinct substrain of TM occurring frequently in patients with inflammatory anogenital tinea.^15^

TMVII is distinguished from other *Trichophyton mentagrophytes* genotypes by sequencing the internal transcribed spacer (ITS) region. In fungal culture, TMVII can also be identified based on characteristic phenotypic features, including rapid growth and dark pigmentation on the underside of colonies, in addition to the typical morphology of *T. mentagrophytes*.^16^, ^17^ Microscopic examination may offer additional clues, such as fewer or absent macroconidia and more frequently occurring chlamydospores compared to other TM strains. In our mycological laboratory, culture‐based phenotypic identification has been consistently applied in routine diagnostics and internally validated against ITS sequencing.

Since its genetic characterization, TMVII has been increasingly identified in patients with genital, perianal, and perioral tinea.^17^ Scientific reports suggest endemic and anthropogenic transmission in multiple regions: Thirty‐seven cases of inflammatory pubogenital TMVII infection were identified between 2016 and 2017 in male and female patients in Berlin, most of whom had no history of travel abroad or animal contact.^15^ In Paris, 13 cases were described with lesions in the beard, genital, and gluteal areas, primarily in men who have sex with men (MSM), suggesting transmission within sexual networks.^3^ More recently, further cases among MSM have been documented in New York,^18^, ^19^ Paris,^20^ and Barcelona.^21^

Clinically, tinea corporis typically presents as superficial, erythematous, scaly plaques with marginal accentuation, resembling a classical “ringworm” morphology.^22^ However, TMVII infections frequently deviate from this pattern, presenting with a markedly inflammatory and purulent morphology.^3^, ^15^, ^17^, ^23^ Apart from atypical clinical presentations, the unusual anatomical localization can complicate and delay diagnosis of this dermatophytosis.^3^ Moreover, the treatment often requires prolonged systemic antifungal therapy and early termination of treatment can lead to recurrence.

Data on the epidemiological and clinical characteristics and available treatment options are scarce. This study intends to provide information on epidemiological characteristics, clinical features, disease course, and treatment outcomes of TMVII infections.

## PATIENTS AND METHODS

### Study design, setting, and ethical considerations

This non‐interventional cohort study included patients with culture‐confirmed TMVII infection treated at the Department of Dermatology, Venereology and Allergology, Charité – Universitätsmedizin Berlin, between January 2020 and August 2024. Patients seen before July 2023 were included retrospectively. Data were extracted from the medical records. From July 2023 onward, patients who provided written informed consent were enrolled prospectively and additionally underwent structured interviews. Ethics approval for patient inclusion was obtained from the institutional review board (EA4/209/23).

### Participants

Patients were eligible if they had a culture‐based, morphologically confirmed TMVII infection and were aged 18 years or older. For fungal culture, skin scales, depilated hair shafts, or pus from lesions were inoculated onto modified dermatophyte agar plates (Sifin, Berlin, Germany; composition per liter: glucose 20.0 g, chloramphenicol 0.1 g, cycloheximide 0.4 g, peptone 10 g, agar 16 g) and incubated for at least 4 weeks. TMVII diagnosis was based on macro‐ and microscopic phenotypic evaluation, as described in the introduction.

### Variables

**FIGURE 1:**
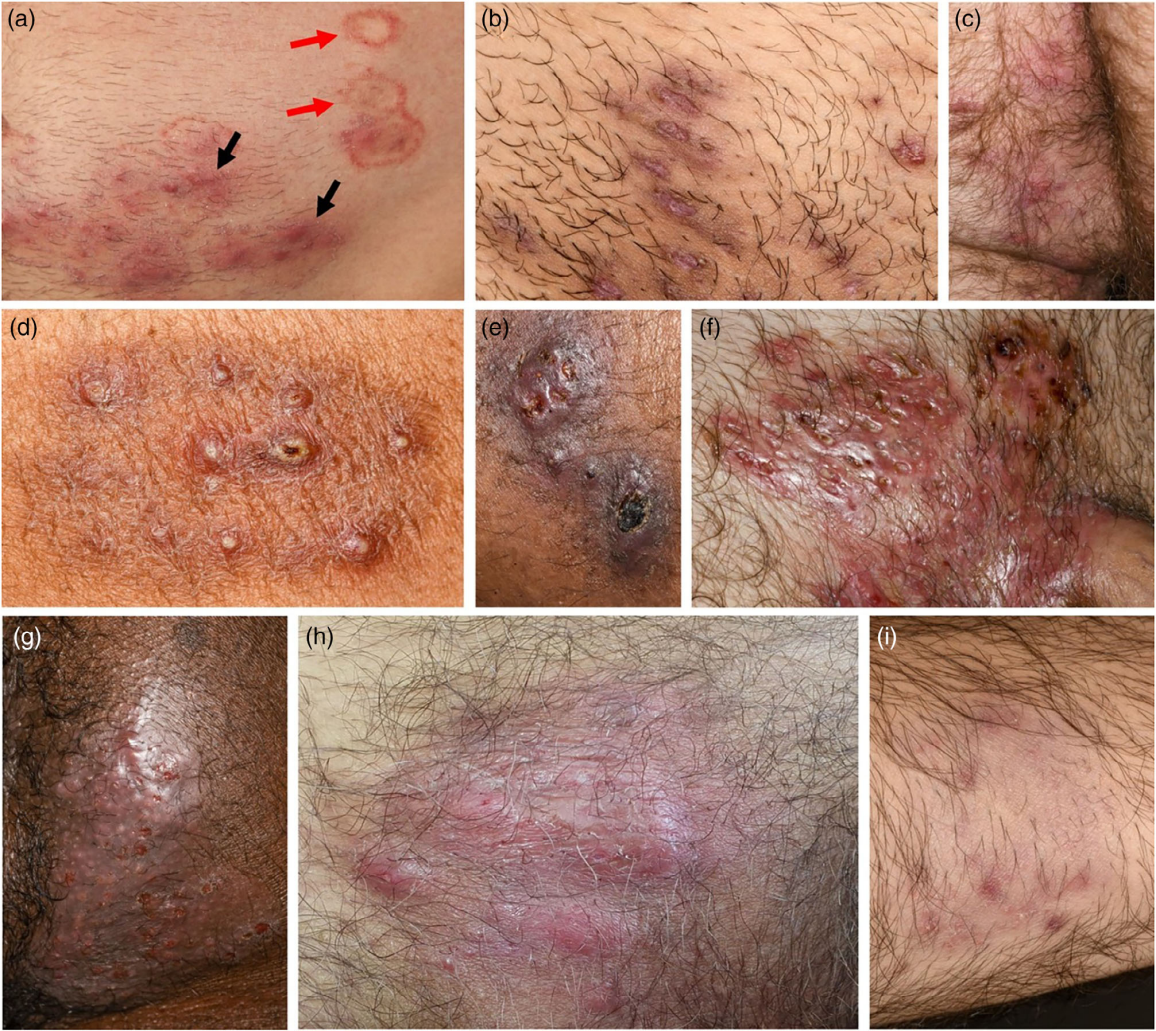
Clinical presentations of Trichophyton mentagrophytes type VII infections. (a) Typical tinea corporis lesions (superficial, well‐demarcated erythematous plaques with marginal accentuation and scaling; red arrows) and atypical subcutaneous papules and pustules (black arrows) on the pubic area and lower abdomen. (b–d) Subcutaneous papules and pustules, partly follicular, in the pubic, perianal, and gluteal regions of three different patients. (e–h) Confluent subcutaneous pustules and papules forming nodules and plaques. (i) Localized alopecia on the forearm.

We collected data on patient characteristics, clinical features, disease course, treatment, and sequelae. Patient characteristics included age, gender, sexual preference, partnership status, number of sexual partners, animal contact, and the patient's suspected infection mode. Additionally, HIV status, use of HIV pre‐exposure prophylaxis (PrEP), immunosuppression, and history of sexually transmitted infections (STIs) were recorded. Clinical features included pain and itch, rated on a numeric rating scale (NRS) from 0 to 10, anatomic localization, lesion morphology (Table 1, Figure 1), and regional lymphadenopathy. Disease course included time from symptom onset to either diagnosis or start of systemic antifungal treatment (whichever occurred first), overall duration of systemic treatment (including medication switches but excluding interruptions), and duration of the first treatment episode (for patients who resumed systemic treatment after a break). Treatment data covered the type, sequence, and combination of systemic and topical antifungal therapies. At the last follow‐up, sequelae such as scarring, hyperpigmentation, pain, and itch were assessed.

**TABLE 1:** Classification of lesion morphology.

Morphology	Description	Classification
Superficial	Superficial erythematous macules or plaques with marginalized accentuation and scaling (example, see Figure 1a, red arrows)	Typical tinea corporis morphology
Purulent	Purulent lesions, e.g., follicular pustules (Figure 1d) or purulent plaques (Figure 1g)	Atypical morphology
Subcutaneous	Subcutaneous, deeply infiltrated papules (Figure 1a, black arrows), nodules (Figure 1h) or plaques (Figure 1h, g)	Atypical morphology

### Data sources/measurement

Patients were identified through a systematic review of case files for retrospective inclusion (until June 2023) and by outpatient clinic physicians for prospective inclusion (from July 2023 onwards). Data were collected from medical records. Additionally, structured interviews were conducted with the prospectively included patients to obtain comprehensive and standardized information on the variables detailed above. To ensure confidentiality, all participants were assigned pseudonyms, which were stored in a password‐protected, encrypted file separate from the study data.

### Data analyses

Categorical variables were summarized as frequencies and percentages. Continuous variables were described using means and standard deviations or medians and quartiles (Q1, Q3), depending on data distribution. Distribution of numerical data was assessed using box plots, histograms, and the Kolmogorov‐Smirnov tests. Fisher's exact test was used to evaluate associations between categorical variables; to quantify the strength of association between anatomic localization and lesion morphology with subjective symptoms and sequelae, risk ratios (RR) and corresponding 95% confidence intervals (95% CI) were calculated. Mann‐Whitney U and Kruskal‐Wallis tests were used to assess differences in the diagnosis and treatment duration based on morphology and HIV/PrEP status. Statistical analyses were conducted using R with RStudio (version 2024.04.2+764). We set the significance threshold at α = 0.05 and applied the Benjamini‐Hochberg (BH) procedure to control the false discovery rate; both unadjusted and BH‐adjusted p values are reported.^24^, ^25^

## RESULTS

### Demographic characteristics

We included 51 patients, 34 of these retrospectively and 17 prospectively. Ages ranged from 24 to 69 years (mean: 37.2, standard deviation [SD]: 11.2; median: 34, Q1–Q3: 29.5–40). Forty‐nine patients (96.1%) were male. Information on sexual orientation was available for 37 male patients, with 35 of these (94.6%) reporting having sex with men and two (5.4%) indicating having sex with women only. Both women indicated having sex with men. Demographic characteristics are shown in Table 2.

**TABLE 2:** Demographic characteristics of the participants.

**Age (years), n = 51**		
Median (Q1–Q3)	34	(29.5–40)
Range	24–69	
**Gender, n = 51**		
	***n***	***%***
Cis male	49	96.1
Cis female	2	3.9
**Sexual orientation, n = 39**		
	*n*	*%*
MSW	2	5.1
MSM	34	87.2
WSM	2	5.1
MSMW	1	2.6
**Number of sex partners (six months prior to the start of the symptoms), n = 22**		
Median (Q1–Q3)	7	(1.0–21.25)
Range	1–100	

*Abbr*.: Q1, first quartile; Q3, third quartile; MSW, men who have sex with women; MSM, men who have sex with men; WSM, women who have sex with men; MSMW, men who have sex with men and women

Of the 47 patients with information on HIV status, nine (19.1%) were living with HIV (PLWH). One of these patients was newly diagnosed in the context of the present fungal infection. This patient had a detectable viral load and a CD4‐count of 280/µl, while all other PLWH had an undetectable viral load. Among 38 HIV‐negative patients, 27 (71.1%) indicated using PrEP. Apart from HIV, no known immunosuppression was present at the time of the diagnosis.

The number of sex partners in the 6 months prior to the start of symptoms ranged from one to 100 (median: 7, Q1–Q3: 1–21.25, n = 22). Among the 31 patients who provided information on their history of STIs, 29 (93.5%) reported having had an STI at some point. A life‐time diagnosis of chlamydia, gonorrhea, syphilis, and hepatitis C was reported by 65.4%, 59.3%, 56.7%, and 3.8%, respectively. A median of one STI diagnosis in the 12 months before diagnosis of the fungal infection was reported (range 0–6, Q1–Q3: 1–2, n = 26). There was a correlation between number of sex partners and STI diagnoses, but this was not significant after BH‐adjustment (*p* = 0.601, *p* = 0.008, BH‐adjusted *p* = 0.088, n = 18).

Four participants reported having had a sex partner with similar lesions or a known fungal infection. Eight of 28 (28.6%) reported having had contact with dogs or cats, though only one of them had observed erythematous patches on their dog.

### Clinical presentation of the acute infection

Twenty‐four patients (47.1%) had a single anatomical region affected. Fifteen (29.4%), eight (15.7%), and four (7.8%) patients had two, three, or four anatomical regions involved, respectively. Remarkably, 49 patients (96.1%) had infections localized on the perianal, genital and/or facial regions. Further details on the anatomical distribution of lesions are shown in Table 3.

**TABLE 3:** Clinical presentation of the lesions.

**Localization, n = 51**	**n**	**%**
Genital, perianal, and/or facial region involved	49	96.1
Genital, pubic area	27	52.9
Perianal, gluteal	18	35.3
Perioral, face	25	49.0
Scalp	4	7.8
Extremities/trunk	2	3.9

**Morphology, n = 51**	**n**	**%**
Superficial lesions	40	78.4
Superficial lesions only	9	17.6
Atypical morphology	42	82.4
Purulent lesions	40	78.4
Subcutaneous lesions	14	27.5
Atypical lesions only	11	21.6

**Lymphadenopathy, n = 28**	**n**	**%**
Present	9	32.1
Not present	19	67.9

Of the 51 patients, only nine (17.6%) presented with exclusively superficial lesions. Forty‐two patients (82.4%) had atypical presentations of tinea involved, such as purulent lesions (40 cases, 78.4%) and/or subcutaneous nodi, plaques, or infiltrates (14 cases, 27.5%). Eleven patients (21.6%) had an atypical presentation only. Further details on clinical presentations and examples are shown in Table 3 and Figure 1.

Purulent lesions occurred more frequently among participants using PrEP (24 out of 27, 88.9%) or with a known HIV infection (8 out of 9, 88.9%) than in the other participants (5 out of 11, 45.5%), although this association was not significant after BH‐adjustment (Fisher's exact test p = 0.013, BH‐adjusted p = 0.072). Otherwise, morphology did not significantly differ according to HIV status or PrEP use.

Subjective symptoms are shown in Table 4. Pain was reported by 31 of 39 (79.5%). Median pain NRS score was 7 (Q1–Q3: 4–8, range 1–8, n = 17). Thirty out of 36 patients (83.3%) reported itch, with a median NRS score of 7 (Q1–Q3: 4–8, range 2–10, n = 18). Although patients with atypical morphology reported pain more frequently (87.1%) than those with superficial lesions only (50.0%), this difference was not statistically significant (RR = 1.74, 95% CI [0.86–3.53]). Likewise, the proportion of patients experiencing pain was higher among those who had anogenital lesions, but there was no significant association between anogenital localisation and pain (RR = 1.25, 95% CI: 0.77–2.04). There were no differences in pain or itch frequency according to HIV status or PrEP use (pain: p = 0.86, BH‐adjusted p > 0.99, itch: p > 0.99, BH‐adjusted p > 0.99, Fisher's exact test).

**TABLE 4:** Subjective symptoms.

**Pain, n = 39**	**n**	**%**
Pain present	31	79.5
Pain, numeric rating scale (0–10), n = 17		
Median (Q1–Q3)	7	(4–8)
Range	1 – 8	

**Itch, n = 36**	**n**	**%**
Itch present	30	83.3
**Itch, numeric rating scale (0–10), n = 18**		
Median (Q1–Q3)	7	(4–8)
Range	2–10	

*Abbr*.: Q1, first quartile; Q3, third quartile

Median time from first symptoms to confirmation of the fungal infection or the initiation of a systemic antimycotic treatment was 42.5 days (Q1–Q3: 29.25–73.5, range 12–294 days, n = 48). This did not differ significantly between patients who had purulent or subcutaneous lesions and those who had superficial lesions only (p = 0.15, BH‐adjusted p = 0.34, Mann‐Whitney U Test) nor between PrEP users, PLWH and other participants (p = 0.44, BH‐adjusted p = 0.80, Kruskal‐Wallis‐Test). The duration of diagnosis correlated moderately positive with age (ρ = 0.35, p = 0.014, BH‐adjusted p = 0.051, n = 48) and moderately negative with the number of anatomic localizations affected (ρ = ‐0.35, p = 0.015, BH‐adjusted p = 0.041, n = 48).

### Treatment and course of the disease

All but one patient (98%) received topical treatment, either additionally to their systemic treatment or as their only treatment. Twenty‐six patients (52.0%) received one topical substance, while 24 (48.0%) received more than one topical substance, either in combination or in sequence. Forty‐one patients (82.0%) were prescribed ciclopirox olamine, 12 (24.0%) ketoconazole, 11 (22.0%) miconazole, 10 (20.0%) clotrimazole, 2 (4.0%) econazole, and 1 (2.0%) topical terbinafine.

Systemic treatment was initiated in 46 of 49 patients (93.9%). While 37 of the 46 systemically treated patients (80.4%) received only one systemic drug, 9 (19.6%) had two or more substances used in sequence. Substances used to initiate systemic treatment were terbinafine, itraconazole, and fluconazole in 38 (77.6%), 7 (14.3%), and 1 (2.0%) of the patients, respectively. Four out of 38 patients (13.2%) who initiated treatment with terbinafine were switched to itraconazole (suspected treatment‐related adverse events (AEs) in 2 cases, insufficient response to treatment in 2 cases), and one was switched to fluconazole by an external physician due to insufficient response after a short course of terbinafine (unclear duration). The latter patient responded adequately after treatment with terbinafine was re‐initiated. Three out of 7 patients who initiated treatment with itraconazole (42.9%) were switched to terbinafine because of an insufficient response. The only patient initiated on fluconazole treatment was switched to itraconazole due to insufficient response after a 42‐day treatment period. AEs were documented for 8 patients (17.4%), as summarized in Table 5.

**TABLE 5:** Adverse events (AEs).

**Total number of AE**	**8**	**Under treatment with …**
Liver enzymes elevation	1	Terbinafine
Gastrointestinal adverse events	3	Terbinafine
Hypogeusia	1	Terbinafine
Daytime fatigue	1	Terbinafine
Drug eruption^*^	1	Terbinafine
Phototoxic reaction^*^	1	Terbinafine

* Discontinuation/switch of therapy

The median overall duration of the systemic treatment, excluding treatment breaks, was 83.5 days (Q1–Q3: 57.0–110.25, range 28–226 days, n = 40). There were no significant differences in the duration of the systemic treatment when comparing patients who only had superficial lesions with those who had purulent or subcutaneous lesions (p = 0.69, BH‐adjusted p = 0.95, Mann‐Whitney U Test) or according to HIV status or PrEP use (p = 0.88, BH‐adjusted p = 0.97, Kruskal–Wallis Test). Systemic treatment was interrupted in nine cases; the reason for the interruption was an initial clinical cure with recurrence in six cases. In these patients, the median duration of the first treatment episode was 42 days (Q1–Q3: 34.5–95.5, range 27–122 days). Overall, the median time between symptom onset and last follow‐up was 152 days (Q1–Q3: 93.5–295.5, n = 37).

### Sequelae at follow‐up

The median time between the end of the systemic treatment and the last follow‐up was 4.5 days (Q1–Q3: ‐3.5–45, n = 34). Data on the clinical appearance and sequelae at the end of or after treatment were available from 34 patients. Twenty‐five of these (73.5%) still had clinically apparent skin changes, with post‐inflammatory hyperpigmentation in 13 (38.2%), erythema in nine (26.5%), and scars or alopecia in six (17.7%) patients. One out of 24 (4.2%) reported itch and none reported pain.

Compared with those who had superficial lesions only, the risk of skin sequelae was slightly higher, although without statistical significance, in patients who had purulent or subcutaneous lesions, (RR: 1.36, 95% CI: 0.69–2.67). There were no clinically or statistically significant differences in the presence of skin sequelae according to HIV status or PrEP use (p = 0.49, BH‐adjusted p = 0.77, Fisher's exact test).

## DISCUSSION

This non‐interventional cohort study presents epidemiological and clinical data from patients with culture‐confirmed TMVII infection treated at a large university medical center in Berlin. By reporting on patient characteristics, clinical presentation, disease course, and treatment outcomes, we aimed to contribute to the limited body of evidence on this emerging fungal pathogen. The findings from our cohort support the growing evidence of endemic spread, with sexual transmission playing a predominant role. Our data underscore the frequent occurrence of atypical clinical presentations, including purulent and subcutaneous lesions, and the prolonged treatment durations often required to achieve mycological clearance.

As seen in recent case series,^3^, ^18^, ^19^ TMVII was primarily acquired by sexually active MSM. Besides the anatomical localization of the lesions, with more than 95% of the participants exhibiting lesions near mucous membranes, several highly prevalent epidemiological characteristics further support sexual transmission. These include a history of multiple sex partners, frequent past STIs, and a high proportion of patients using PrEP or living with HIV. Although TMVII infections remain far less prevalent than other STIs, their epidemiological pattern among MSM mirrors trends observed in other STIs, such as syphilis or mpox.

In addition to behavioral and network‐related factors, such as participation in sexual networks with a high epidemiological burden of STIs, the high proportion of MSM with multiple partners and frequent history of STIs could also point towards increased biological susceptibility. Potential contributing factors include microtrauma to the skin and alterations in the skin microbiome due to frequent antibiotic use. Host‐pathogen interactions in the context of sexually transmitted fungal infections remain poorly understood. The observed association with HIV and PrEP use may, in part, also reflect increased engagement with sexual health services, potentially leading to earlier detection of TMVII infections. However, given the high proportion of patients experiencing relevant complaints, it is likely that most affected individuals would have sought medical care regardless of background.

In line with previous reports on TMVII infections,^3^, ^15^, ^17^, ^23^ our cohort confirms that a high proportion of patients presented with clinically atypical manifestations of tinea corporis, including purulent lesions involving hair shafts as well as deeply infiltrated subcutaneous plaques and nodules – distinct from the typical superficial “ringworm” lesions characterized by marginal erythema and scaling. This might be a consequence of the close genetic proximity of TMVII with other TM genotypes of zoophilic origin.^15^ An intensely inflammatory reaction on fungal infections is typical for zoophilic dermatophytes when affecting humans.^1^ The atypical presentation may delay proper diagnosis and treatment. While the median time from symptom onset to diagnosis was just under 1.5 months, 25% of patients experienced a diagnostic delay of more than 73 days, with one case extending to 290 days.

Our nearly five‐month median follow‐up indicates that the infection affected many patients over an extended period, highlighting treatment challenges. In superficial tinea corporis caused by other dermatophytes, systemic treatment usually lasts 2 to 4 weeks. The median treatment duration in our cohort of patients was 12 weeks, with a quarter of patients requiring more than 15 weeks. This is consistent with the findings of a case series from Paris, in which systemic therapy was likewise administered for up to four months.^3^ In a small proportion of patients who received treatment for a median duration of only 42 days, recurrence was observed, necessitating re‐initiation of therapy.

We found no significant differences in treatment duration between atypical and typical clinical presentations. However, due to the small number of patients with superficial lesions only, this comparison was likely underpowered. The high proportion of patients with subcutaneous and purulent lesions is a plausible explanation for the prolonged treatment duration observed in our cohort. Another potential contributing factor could be a rise in terbinafine resistance among dermatophytes. Although widespread terbinafine resistance is currently most often associated with *Trichophyton indotineae* (formerly termed TM genotype VIII),^26^, ^27^, ^28^, ^29^ cases of reduced susceptibility have also been reported in other *Trichophyton* species. In our cohort of patients, only two out of 38 patients initially treated with terbinafine were switched to itraconazole because of suspected treatment failure. While this suggests that terbinafine resistance was not a major factor in our population, reduced antifungal susceptibility and the potential for emerging resistance in TMVII should not be overlooked and warrants further investigation. Ongoing molecular surveillance of resistance‐associated mutations may be important to detect shifts in susceptibility early.^30^

No significant differences in treatment duration or efficacy were seen between PLWH and PrEP users. Notably, all but one of the PLWH in our cohort were under antiretroviral treatment and had well‐controlled infection with undetectable viral loads, which may explain the comparable treatment outcomes. Although the analysis of potential confounders was limited by the relatively low number of PLWH, we did not identify any other plausible factors that could explain the lack of association between HIV status and treatment response.

A limitation of our study is that the diagnosis of TMVII infections relied on phenotypic evaluation of fungal cultures, as outlined in the Introduction. Although ITS sequencing remains the gold standard for species identification and genotyping,^17^, ^31^ the specific morphological characteristics of TMVII on cycloheximide‐containing dermatophyte agar or common Sabouraud dextrose agar have been documented in previous publications.^16^, ^17^ In our mycological laboratory, the culture‐based phenotypic approach has been consistently applied and internally validated in 31 prior isolates. In all of these cases, isolates exhibiting characteristic macro‐ and microscopic features consistent with TMVII were confirmed by ITS sequencing, with no discordant results. This supports the reliability of our culture‐based phenotypic approach; however, its diagnostic accuracy has not yet been systematically assessed. Therefore, the possibility of diagnostic misclassification cannot be entirely excluded. Notably, the epidemiological and clinical characteristics of the included patients were highly consistent and align closely with those reported in international studies where TMVII was confirmed by ITS sequencing.^18^, ^19^, ^20^, ^21^ This further supports the validity of our culture‐based diagnostic approach. Although further research is needed to establish its diagnostic reliability, the culture‐based phenotypic identification may provide a cost‐effective alternative to distinguish TMVII from other TM genotypes.

It is also important to consider that the representativeness of our findings may be limited due to a potential selection toward severe or refractory cases, given that recruitment took place at a university hospital. Although we have identified potential risk factors, the small sample size may not allow for the extrapolation of relevant conclusions. Future studies should investigate differences between atypical and typical lesions in terms of patient characteristics and treatment response.

It is important to incorporate TMVII into the differential diagnosis for genital, perianal, and perioral skin lesions, particularly among at‐risk populations such as MSM with multiple partners. Raising awareness of this emerging pathogen among clinicians and affected communities is necessary to enable timely diagnosis, informed counseling and effective treatment. Improved knowledge of its epidemiological and clinical characteristics can support appropriate case management and contribute to infection control efforts.

## CONFLICT OF INTEREST STATEMENT

None.
